# The enigmatic linguistic cerebellum: clinical relevance and unanswered questions on nonmotor speech and language deficits in cerebellar disorders

**DOI:** 10.1186/2053-8871-1-12

**Published:** 2014-09-01

**Authors:** Peter Mariën, Alan Beaton

**Affiliations:** Clinical and Experimental Neurolinguistics, CLIN, Vrije Universiteit Brussel, Pleinlaan 2, 1050 Brussels, Belgium; Department of Neurology and Memory Clinic, ZNA Middelheim General Hospital, Lindendreef 1, 2020 Antwerp, Belgium; Department of Psychology, Swansea University, Swansea, Wales UK; Department of Psychology, Aberystwyth University, Aberystwyth, Wales UK

**Keywords:** Cerebellum, Language, Speech, Apraxia of speech, Verbal fluency, Syntax, Phonology, Semantics, Aphasia, Agraphia, Dyslexia, Functional topography, Imaging, fMRI, SPECT

## Abstract

Clinical case descriptions and experimental evidence dating back to the early part of the 19th century from time to time documented a range of nonmotor cognitive and affective impairments following cerebellar pathology. However, a causal relationship between disruption of nonmotor cognitive and affective skills and cerebellar disease was dismissed for several decades and the classical view of the cerebellum as a mere coordinator of autonomic and somatic sensorimotor function prevailed for more than two centuries in behavioural neuroscience. The ignorance of early clinical evidence suggesting a much richer and complex role for the cerebellum than a pure sensorimotor one is remarkable given that in addition: 1) the cerebellum contains more neurons than the rest of the combined cerebral cortex and 2) no other structure has as many connections with other parts of the brain as the cerebellum. During the past decades, the long-standing view of the cerebellum as pure coordinator of sensorimotor function has been substantially modified. From the late 1970s onwards, major advances were made in elucidating the many functional neuroanatomical connections of the cerebellum with the supratentorial association cortices that subserve nonmotor language, cognition and affect. Combined with evidence derived from experimental functional neuroimaging studies in healthy subjects and neurophysiological and neuropsychological research in patients, the role of the cerebellum has been substantially extended to include that of a crucial modulator of cognitive and affective processes. In addition to its long-established role in coordinating motor aspects of speech production, clinical and experimental studies with patients suffering from etiologically different cerebellar disorders have identified involvement of the cerebellum in a variety of nonmotor language functions, including motor speech planning, language dynamics and verbal fluency, phonological and semantic word retrieval, expressive and receptive syntax processing, various aspects of reading and writing and aphasia-like phenomena. Despite considerable efforts currently devoted to further refine typology and anatomoclinical configurations of nonmotor linguistic dysfunctions linked to cerebellar pathology, the exact underlying pathophysiological mechanisms of cerebellar involvement remain to be elucidated.

## Introduction

For more than two centuries clinical and experimental research on the cerebellum has been overshadowed by an overwhelming interest in the role of the cerebellum in sensorimotor control including diadochokinesia, tonus, coordination, and motor aspects of speech production [1, 2]. A wealth of experimental and clinical evidence exists to support the view that the cerebellum primarily coordinates movement, resulting in various cerebellar ataxic syndromes in cases where the motor zones of the cerebellum incur neurological damage. However, during the past decades, the long-standing view of the cerebellum as pure coordinator of sensorimotor function has been substantially modified. From the late 1970s onwards, major advances were made in elucidating the many functional neuroanatomical connections of the cerebellum with the supratentorial association cortices that subserve nonmotor language, cognition and affect. In addition, experimental functional neuroimaging studies in healthy subjects and neurophysiological and neuropsychological research in patients showed that the cerebellum is critically implicated in a large spectrum of cognitive and affective (dys)functions. As a result, converging evidence derived from these different strands of research substantially extended the sensorimotor role of the cerebellum to include that of a crucial modulator of cognitive and affective processes.

## Review

At the beginning of the 20th century Gordon Holmes [3] not only encouraged the clinical investigation of cerebellar pathology, he also put forward the view that the cerebellum plays a crucial role in *motor speech production*. His classic paper on the effects of gunshot wounds in 21 victims of world war I described ‘decomposition of movement’ affecting the muscular control of speech, closely anticipating the authoritative auditory-perceptual investigation of the Mayo Clinic that followed several decades later. Holmes [3] wrote:

‘(Speech) is usually slow, drawling and monotonous, but at the same time tends to be staccato and scanning. This gives it an almost typical “sing-song” character and makes it indistinct and often difficult to understand (…) In many cases the utterance is remarkable irregular and jerky, and that of many syllables (…) tends to be explosive. Phonation is as a rule more affected than articulation, though both vowels and consonants are slurred and uttered unequally and irregularly. All classes of consonants too are affected, but articulation sometimes has a special nasal character and the labials particularly tend to be explosive. Another striking feature is the apparent effort necessary to utter a series of syllables or a sentence; the attempt is associated with excessive facial grimacing and speech has consequently a laboured character that often recalls pseudo-bulbar paresis.’ (p. 505–506).

A number of investigators after Holmes maintained that the responsible lesion for *ataxic dysarthria* could be situated in either one or both cerebellar hemispheres while Lechtenberg and Gilman [4], in a large study of 122 patients, found that dysarthria resulted more frequently from left than right superior cerebellar lesions. Although a laterality effect has not yet been clearly demonstrated, clinical and experimental neuroimaging studies addressing the topographic aspects of motor speech processing have shown that ataxic dysarthria most frequently follows from damage to the (right) superior anterior vermal and paravermal regions, supplied by the superior cerebellar artery (SCA) [5, 6]. As posterior inferior cerebellar artery (PICA) and anterior inferior cerebellar artery (AICA) infarctions are usually associated with brainstem damage, it is not possible to unambiguously attribute motor speech deficits to PICA or AICA lesions [7].

*Apraxia of speech* (AoS) (anarthria, verbal apraxia, speech apraxia) is a motor speech planning and coordination disorder that typically follows from injury to the language dominant motor speech region (anterior insula, inferior premotor and motor cortex, BA 44 of Broca’s area). Characterized by inconsistent misarticulations and irregular articulatory breakdowns, phonetic alterations of vowel and consonant production, dysdiadochokinesis, with sequential errors, flattened voice volume, prosodic abnormalities, slow articulation rate, and scanning of speech, AoS shares a number of overt semiological characteristics with ataxic dysarthria. These similarities may suggest that both conditions represent related phenomena possibly resulting from disruption of the motor speech planning and coordinating network subserved by a close functional interplay between the anterior motor speech region of the language dominant hemisphere and the contralateral right cerebellum. Indeed, AoS, termed *ataxic aphasia* and *cortical dysarthria* in the early literature, has been observed in a number of cases with etiologically heterogeneous cerebellar disorders but a suggested pathophysiological link between AoS and ataxic dysarthria remains to be clarified [8]. In-depth study of the automatico-voluntary dissociations (typically reflecting an underlying apraxic disorder) and the motor speech characteristics of patients recovering from cerebellar mutism (anarthria) in the context of posterior fossa syndrome, might, for instance, help to unravel the presumed role of the cerebellum in motor speech planning disorders [8–10].

*Impaired verbal fluency* is a common finding in patients affected by focal or degenerative lesions of the cerebellum [2, 11]. Despite a lack of agreement regarding the laterality of cerebellar involvement, a number of clinical and neurophysiological studies have shown that in patients with cerebellar pathology forced phonemic word retrieval from the lexicon (phonological fluency) is more affected than the ability to generate words according to a semantic rule (semantic fluency) [12]. It remains to be shown whether the disruption of sequencing processing as a general mechanism subserved by the cerebellum might account for a disruption of *language dynamics* at other linguistic levels as well ((morpho) syntax, pragmatics) resulting in dynamic or a transcortical-like aphasia, even to the point of mutism due to inhibition of self-generated speech and language production [13].

Although current insights into the role of the cerebellum in *syntax processing* are still limited, evidence derived from clinical, neurophysiological and neuroimaging studies demonstrates that the cerebellum contributes to grammar processing, in both the expressive and receptive domain [14]. In a much cited study introducing the concept of ‘cerebellar cognitive affective syndrome’ (CCAS), Schmahmann and Sherman included agrammatism as part of the cluster of linguistic disturbances following focal cerebellar lesions [15]. Although a few studies indicate that even the left or both cerebellar hemispheres may be involved in grammar processing, most clinical and experimental studies have shown that the right cerebellar hemisphere is embedded within a distinct neural network devoted to processing of grammar, including the basal ganglia and the language dominant left prefrontal, temporal and parietal cortex [16, 17].

In contrast to its *developmental* variant, *acquired dyslexia* (alexia) has scarcely been studied in the context of cerebellar disorders. In a group of 10 patients with (para) vermian lesions, Moretti et al. [18] showed a lower degree of accuracy in reading words and sentences. In comparison to a matched control group, patients made true aphasic reading errors at the letter- and word-level. In a subsequent publication from the same laboratory [19], the reading and writing errors of six patients with olivopontocerebellar atrophy were described. The authors suggested that acquired dyslexia may be related either to an imperfect oculomotor control (nystagmus), or to a disruption of the cerebellar-encephalic projections connecting the cerebellum to the supratentorial areas implicated in language as well as in attentional and alerting processes.

**Figure 1:**
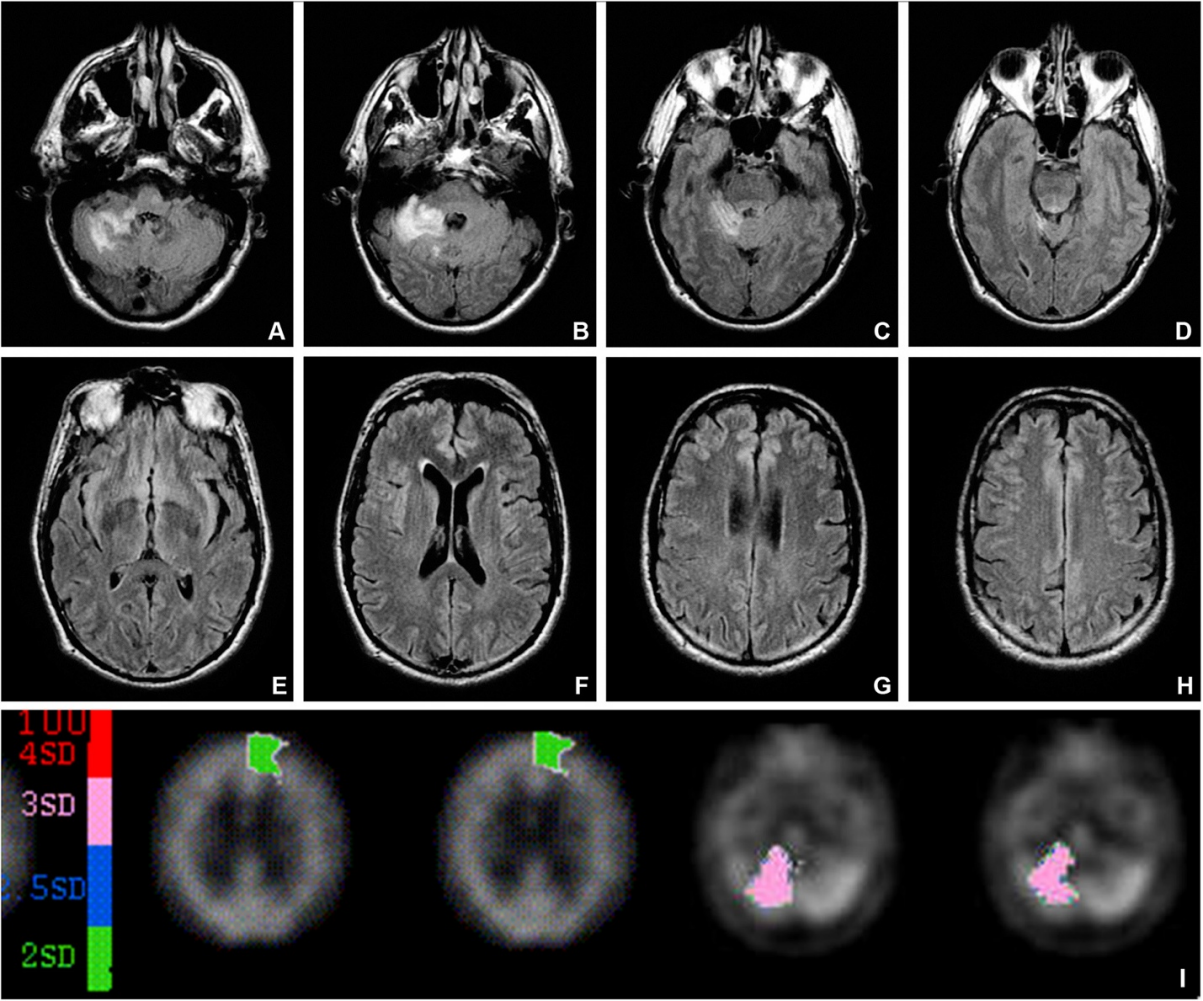
**A-H:** **Brain MRI.** Axial Flair slices showing a right cerebellar-pontine infarction in the vascular territory of the superior cerebellar artery (SCA). No structural damage was observed at the supratentorial level (from [21]). **I: Quantified ECD-SPECT.** SPECT performed 5 weeks post-stroke reveals the phenomenon of crossed cerebello-cerebral diaschisis as reflected by a hypoperfusion in the right cerebellar hemisphere and a distant significant decrease of cerebral blood flow in the left medial frontal area (from [21]).

Very little is known about a possible causal role of the cerebellum in *central agraphia*. Indeed, only a handful of cases exist in which a central agraphia was found after cerebellar damage [20, 21]. Following a right SCA infarction the patient reported by Mariën et al. [21] presented CCAS with *visual dyslexia* and *surface dysgraphia* (Figure 1A-E). As reflected by the phenomenon of crossed cerebello-cerebral diaschisis on SPECT, the authors hypothesized that the linguistic deficits resulted from functional disruption of the cerebellar-encephalic pathways connecting the cerebellum to the frontal supratentorial areas which subserve attentional and planning processes (Figure 1B).

It needs to be clarified whether or not central agraphia following a cerebellar lesion is characterized by a uniform error typology but in all the cases reported so far, distorted written expression was attributed to disruption of integration of cerebellar control in the frontal lobe system. Recently, clinical evidence has been found suggesting involvement of the cerebellum in the neural network devoted to graphomotor planning and execution. *Apraxic agraphia* is a peripheral writing disorder due to distortion of the skilled movement plans that direct the production of letters [22]. Classical tenets posit that the dominant parietal lobe (storage of graphic motor engrams), the dorsolateral premotor cortex and the supplementary motor area (involved in translating these programs into graphic innervatory patterns) constitute the neural network that subserves graphomotor output. However, in addition to thalamic damage apraxic agraphia has recently been associated with functional disruption of the cerebello-cerebral network subserving the planning and execution of skilled motor actions.

The co occurrence of a cluster of linguistic deficits affecting the phonological, lexico-semantic, and syntactic levels to different degrees may give rise to the notion of *cerebellar*-*induced aphasia* [13]. Aphasia-like phenomena following cerebellar lesions may involve the level of speech production, language comprehension, repetition, naming, reading and writing. As supported by the findings of (functional) neuroimaging studies, disruption of linguistic functions after cerebellar lesions may be considered to result from a decrease or loss of excitatory impulses through the cerebello-ponto-thalamo-cortical pathways causing a functional depression of the supratentorial regions that intrinsically subserve linguistic processing in the language dominant hemisphere. Most clinical and experimental studies indicate involvement of a ‘lateralized linguistic cerebellum’ contralateral to the language dominant hemisphere [2, 13].

However, a more skeptical opinion on the role of the cerebellum in linguistic processing has been advanced by Dagmar Timmann and co-workers who address in a number of clinical studies the limitations of lesion studies and negative findings in patients with cerebellar lesions e.g. [23].

**Figure 2:**
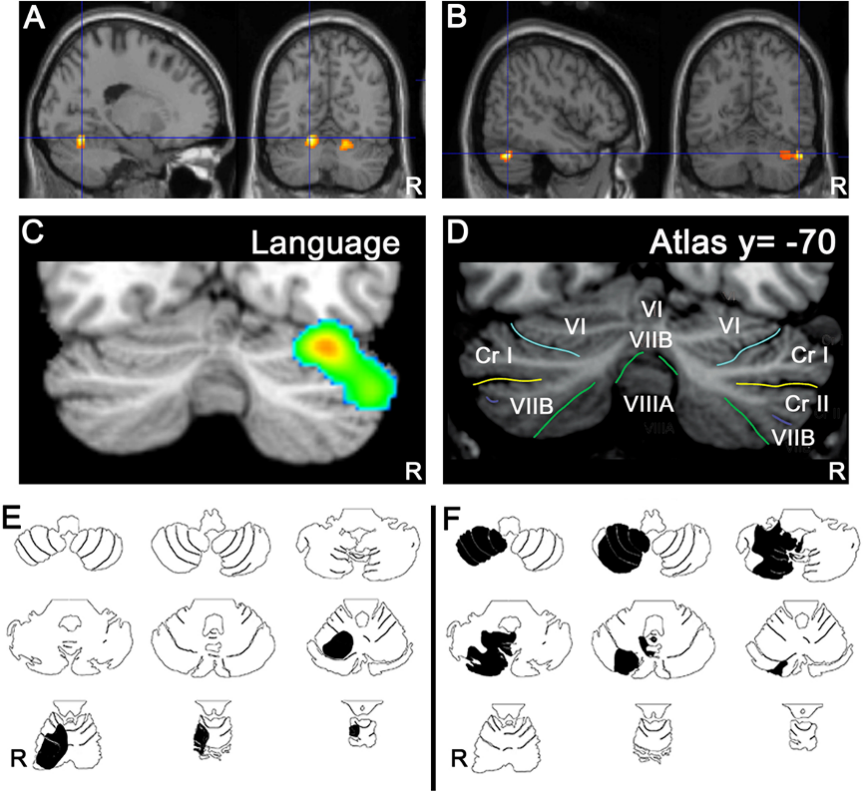
**Topographic arrangement in cerebellum of speech versus language representation.** Functional MRI localizes articulation **(A)** to medial parts of lobule VI bilaterally, whereas verb generation **(B)** activates lateral regions of lobule VI and Crus I on the right. In a meta-analysis of functional imaging studies [24] higher level language tasks engage the right lateral posterior cerebellum, lobules VI and Crus I **(C)** according to the lobule identification in (**D**; [23]). Case studies of cerebellar stroke patients reveal topography for articulation vs. higher-level language tasks. A patient with stroke in the territory of the right superior cerebellar artery (**E**, black shading) involving lobules I-VI was dysarthric; whereas a patient with stroke in the territory of the right posterior inferior cerebellar artery (**F**, black shading) involving lobules VII-IX was not dysarthric but performed poorly on the Boston Naming Test [25, 26] (from [2]).

The neuroanatomical substrate of the role of the cerebellum in nonmotor language processing is the dense and reciprocal network of crossed cerebro-cerebellar pathways consisting of cortico-ponto-cerebellar and cerebello-thalamo-cortical loops that establish a close connection between the cerebellum and the supratentorial motor, paralimbic and association cortices. A plethora of contemporary lesion-behavior and neuroimaging studies has demonstrated that in addition to its somatotopic organization for motor control, the human cerebellum is topographically organized for higher-order cognitive and affective functions. A meta-analysis of neuroimaging studies has provided support for a dichotomy between the sensorimotor cerebellum - geographically organized in distinct regions in the anterior lobe - and the neurocognitive and affective cerebellum - represented in distinct parts in the posterior lobe (for a review see: [2, 24]). In addition, the majority of anatomoclinical studies of patients with linguistic impairments following focal cerebellar lesions and the majority of neuroimaging studies typically show a lateralized involvement of lateral, posterior cerebellar regions (including lobules VI and Crus I/II) in nonmotor linguistic processes (Figure 2).

## Conclusion

In addition to its long-established crucial role in coordinating motor aspects of speech production, an increasing number of clinical and experimental studies with patients suffering from etiologically different cerebellar disorders have identified involvement of the cerebellum in a variety of nonmotor language functions, including motor speech planning, language dynamics and verbal fluency, phonological and semantic word retrieval, expressive and receptive syntax processing, various aspects of reading and writing and even aphasia-like phenomena. Continued theoretically driven clinical and experimental investigations are needed to further improve insights into the functional neuroanatomical network and neurophysiological characteristics of the enigmatic linguistic cerebellum. Substantial refinement of clinical tools designed to capture the often subtle and transient nonmotor language dysfunctions after cerebellar damage is needed to further unravel the linguistic role of the mysterious “little brain” hidden under the cerebral cortex of the occipital lobes.

## References

[1] Manto M, Bower JM, Conforto AB, Delgado-García JM, da Guarda SN, Gerwig M, Habas C, Hagura N, Ivry RB, Mariën P, Molinari M, Naito E, Nowak DA, Oulad Ben Taib N, Pelisson D, Tesche CD, Tilikete C, Timmann D (2012). Consensus paper: roles of the cerebellum in motor control - the diversity of ideas on cerebellar involvement in movement. Cerebellum.

[2] Mariën P, Ackermann H, Adamaszek M, Barwood CHS, Beaton A, Desmond J, De Witte E, Fawcett AJ, Hertrich I, Küper M, Leggio M, Marvel C, Molinari M, Murdoch BE, Nicolson RI, Schmahmann JD, Stoodley CJ, Thürling M, Timmann D, Wouters E, Ziegler W (2014). Consensus Paper: Language and the cerebellum: an ongoing enigma. Cerebellum.

[3] Holmes G (1917). The symptoms of acute cerebellar injuries due to gunshot injuries. Brain.

[4] Lechtenberg R, Gilman S (1978). Speech disorders in cerebellar disease. Ann Neurol.

[5] Ackermann H, Vogel M, Petersen D, Poremba M (1992). Speech deficits in ischaemic cerebellar lesions. J Neurol.

[6] Urban PP, Marx J, Hunsche S, Gawehn J, Vucurevic G, Wicht S, Massinger C, Stoeter P, Hopf HC (2003). Cerebellar speech representation: lesion topography in dysarthria as derived from cerebellar ischemia and functional magnetic resonance imaging. Arch Neurol.

[7] Urban PP (2013). Speech motor deficits in cerebellar infarctions. Brain Lang.

[8] De Smet HJ, Baillieux H, Catsman-Berrevoets C, De Deyn PP, Mariën P, Paquier PF (2007). Postoperative motor speech production in children with the syndrome of ’cerebellar’ mutism and subsequent dysarthria: a critical review of the literature. Eur J Paed Neurol.

[9] Mariën P, Verhoeven J, Engelborghs S, Rooker S, Pickut BA, De Deun PP (2006). A role for the cerebellum in motor speech planning: evidence from foreign accent syndrome. Clin Neurol Neurosurg.

[10] Pitsaka M, Tsitouras V (2013). Cerebellar mutism: a review. J Neurosurg Pediatr.

[11] Leggio M, Silveri M, Petrosini L, Molinari M (2000). Phonological grouping is specifically affected in cerebellar patients: a verbal fluency study. J Neurol Neurosur Ps.

[12] Stuss DT, Alexander MP (2007). Is there a dysexecutive syndrome?. Philos T Roy Soc B.

[13] Mariën P, Saerens J, Nanhoe R, Moens E, Nagels G, Pickut B, Dierckx RA, De Deyn PP (1996). Cerebellar induced aphasia: case report of cerebellar induced prefrontal aphasic language phenomena supported by SPECT findings. J Neurol Sci.

[14] Adamaszek M, Strecker K, Kessler C (2012). Impact of cerebellar lesion on syntactic processing evidenced by event-related potentials. Neurosci Lett.

[15] Schmahmann JD, Sherman JC (1998). The cerebellar cognitive affective syndrome. Brain.

[16] Justus T (2004). The cerebellum and English grammatical morphology: evidence from production, comprehension, and grammaticality judgements. J Cogn Neurosci.

[17] Mariën P, Engelborghs S, Fabbro F, De Deyn PP (2001). The lateralized linguistic cerebellum: a review and a new hypothesis. Brain Lang.

[18] Moretti R, Bava A, Torre P, Antonello RM, Cazzato G (2002). Reading errors in patients with cerebellar vermis lesions. J Neurol.

[19] Moretti R, Torre P, Antonello RM, Carraro N, Zambito-Marsala S, Ukmar MJ, Capus L, Gioulis M, Cazzato G, Bava A (2002). Peculiar aspects of reading and writing performances in patients with olivopontocerebellar atrophy. Percept Mot Skills.

[20] Fabbro F, Tavano A, Corti S, Bresolin N, De Fabritiis P, Borgatti R (2004). Long-term neuropsychological deficits after cerebellar infarctions in two young adult twins. Neuropsychologia.

[21] Mariën P, Baillieux H, De Smet HJ, Engelborghs S, Wilssens I, Paquier P, De Deyn PP (2009). Cognitive, linguistic and affective disturbances following a right superior cerebellar artery infarction: a case study. Cortex.

[22] De Smet HJ, Engelborghs S, Paquier PF, De Deyn PP, Mariën P (2011). Cerebellar-induced apraxic agraphia: a review and three new cases. Brain Cognition.

[23] Frank B, Schoch B, Hein-Kropp C, Dimitrova A, Hövel M, Ziegler W, Gizewski ER, Timmann D (2007). Verb generation in children and adolescents with acute cerebellar lesions. Neuropsychologia.

[24] Stoodley CJ, Schmahmann JD (2009). Functional topography in the human cerebellum: a meta-analysis of neuroimaging studies. Neuroimage.

[25] Schmahmann JD, Doyon J, Toga A, Petrides M, Evans A (2000). MRI Atlas of the Human Cerebellum.

[26] Schmahmann JD, Macmore J, Vangel M (2009). Cerebellar stroke without motor deficit: clinical evidence for motor and non-motor domains within the human cerebellum. Neuroscience.

